# Distinct risk profiles of heart failure and non-heart failure major adverse cardiovascular events among patients with early breast cancer: a competing-risk analysis

**DOI:** 10.1007/s10147-026-03200-1

**Published:** 2026-09-15

**Authors:** Hideo Shigematsu, Atsuyoshi Norishige, Kazuaki Tanabe, Hiroyuki Sawatari, Yukiko Nakano, Ai Amioka, Daiki Okamoto, Sakiko Hino, Haruka Ikejiri, Shinsuke Sasada, Morihito Okada

**Affiliations:** 1 Department of Surgical Oncology, Research Institute for Radiation Biology and Medicine, Hiroshima University, Hiroshima, Japan; 2 Department of Perioperative and Critical Care Management, Hiroshima University, Hiroshima, Japan; 3 Department of Cardiovascular Medicine, Hiroshima University, Hiroshima, Japan; 4 Department of Breast Surgery, Hiroshima University Hospital, Hiroshima, Japan; 5 Department of Surgical Oncology, Research Institute for Radiation Biology and Medicine, Hiroshima University, 1-2-3-Kasumi, Minami-ku, Hiroshima City, Hiroshima 734-8551 Japan

**Keywords:** Anthracyclines, Breast cancer, Major adverse cardiovascular event, Trastuzumab

## Abstract

**Background:**

Cardiovascular complications are increasingly recognized among patients with breast cancer; however, heart failure has been the primary focus of studies to date. We aimed to evaluate the cumulative incidence of heart failure and other major adverse cardiovascular events among patients with early breast cancer and to identify risk factors.

**Methods:**

We conducted a retrospective cohort study of patients with non-metastatic breast cancer who underwent curative surgery at a tertiary center during 2010–2019. The endpoints were heart failure and other major adverse cardiovascular events (stroke, atrial fibrillation, myocardial infarction, or cardiovascular death). The cumulative incidence was estimated using the cumulative incidence function, and risk factors were evaluated using Fine–Gray competing-risk regression models. Breast cancer recurrence and non-cardiovascular event-related death were treated as competing events.

**Results:**

Over a median follow-up of 7.7 years, heart failure occurred in 24/1,328 (1.8%) patients, whereas other major adverse cardiovascular events occurred in 38 (2.9%). The cumulative incidence of heart failure differed according to perioperative systemic therapy (*p* = 0.002). Anthracycline use (subdistribution hazard ratio, 2.67; 95% confidence interval, 1.08–6.65; *p* = 0.034) and trastuzumab use (2.52; 1.00–6.33; *p* = 0.050) were independently associated with heart failure. By contrast, other major adverse cardiovascular events were associated with age (> 65 years; 3.75; 1.59–8.88; *p* = 0.003) and cardiovascular risk factors (4.02; 1.77–9.10; *p* < 0.001).

**Conclusions:**

Heart failure was primarily associated with cardiotoxic cancer therapies, and other events by patient-related cardiovascular risk factors. These findings highlight the importance of integrating cardiotoxicity surveillance with long-term cardiovascular risk management in breast cancer survivorship.

## Introduction

Advances in systemic therapy and radiotherapy have substantially improved the survival of patients with early breast cancer, resulting in a rapidly growing population of long-term survivors [1]. As survivorship increases, late adverse effects of cancer therapy have become increasingly important clinical issues. Among these, cardiovascular complications have emerged as a major concern because they may markedly affect patients’ long-term prognosis and quality of life [2]. Accordingly, the field of cardio-oncology has evolved for the cardiovascular risk assessment, surveillance, and multidisciplinary management of patients undergoing breast cancer treatment [3].

Several breast cancer therapies are known to cause cardiotoxicity. Anthracyclines induce dose-dependent myocardial injury that may lead to heart failure (HF), while anti-human epidermal growth factor receptor 2 (HER2) therapies such as trastuzumab have proven to be associated with left ventricular dysfunction and HF in both clinical trials and real-world studies [4–6]. These treatment-related cardiac effects have been widely studied, and clinical guidelines emphasize the need for careful cardiac monitoring before and during therapy [7].

However, cardiovascular adverse events caused by breast cancer treatment are not limited to HF. Other clinically important events—including stroke, myocardial infarction (MI), atrial fibrillation (AF), and cardiovascular disease-related death—may also occur during long-term follow-up [8–10]. Although cardiovascular events in patients with breast cancer have been evaluated in several large population-based studies, the definitions and components of major adverse cardiovascular events (MACEs) have varied substantially across them, complicating direct comparisons [11]. Furthermore, recurrence of breast cancer and non-cardiovascular event-related death may act as competing events in the evaluation of cardiovascular outcomes, yet researchers have not explicitly accounted for these competing risks in relevant studies to date [12, 13].

In Japan, most studies evaluating cardiovascular complications in patients with breast cancer have primarily focused on heart failure or left ventricular dysfunction, particularly in the context of trastuzumab-associated cardiotoxicity [14–16].

Therefore, in this study, we aimed to evaluate the cumulative incidence of HF and non-HF MACEs among patients with early breast cancer. We also aimed to identify clinical and treatment-related risk factors—including anthracycline use, trastuzumab use, radiotherapy, and patient comorbidities—via a competing-risk framework.

## Patients and methods

### Study population

This was a single-center, retrospective cohort study. Patients who were diagnosed with breast cancer and subsequently underwent curative surgery at the Department of Breast Surgery, Hiroshima University Hospital from 2010 to 2019 were identified from their electronic medical records and the institutional database. Patients with ductal carcinoma in situ, which is typically not treated with cardiotoxic systemic therapy; those with a history of cardiovascular disease prior to the breast cancer diagnosis, including HF, AF, MI, or stroke; and those for whom essential clinical data were missing were excluded. Follow-up began on the date of breast cancer diagnosis. Cardiovascular events, breast cancer recurrence, and death were identified through review of electronic medical records. Follow-up was administratively censored at 10 years after diagnosis to reduce potential bias arising from disproportionately longer follow-up in a subset of patients, which could lead to an overrepresentation of events in the late follow-up period. This study was approved by the Institutional Review Board of Hiroshima University (approval number: E2024-0224). Given the retrospective design and the use of data obtained from electronic medical records with appropriate protection of patient privacy, the requirement for written informed consent was waived by the Institutional Review Board.

### Outcomes and definitions

Because no standardized composite endpoint for cardiovascular events has been established in cardio-oncology, we predefined non-heart-failure MACE as the first occurrence of stroke, myocardial infarction, newly diagnosed atrial fibrillation, or cardiovascular death. This definition was selected to capture the major clinically relevant cardiovascular complications recognized in contemporary cardio-oncology, while evaluating heart failure separately [7]. In particular, atrial fibrillation was included because recent cardio-oncology guidelines recognize it as one of the major cardiovascular complications associated with cancer and anticancer therapy, with important implications for subsequent stroke, heart failure, and mortality [7]. Stable angina, unstable angina requiring hospitalization, and coronary revascularization procedures were not included because they represent therapeutic interventions or manifestations of coronary artery disease rather than predefined cardiovascular outcome events in the present study. Furthermore, unlike conventional cardiovascular outcome trials in which heart failure is often defined as hospitalization for heart failure, heart failure in this study was defined according to contemporary cardio-oncology recommendations as clinically diagnosed heart failure or clinically significant left ventricular systolic dysfunction (LVEF < 50%), reflecting cancer therapy-related cardiac dysfunction [7, 17].

### Clinicopathological variables

Age was dichotomized at 65 years for clinical interpretability [18]. Cardiovascular risk factors were defined as the presence of hypertension, diabetes mellitus, or dyslipidemia. Clinical stage was classified according to the American Joint Committee on Cancer staging system, and cases were grouped as stage I or stage II–III [19]. Tumor subtypes were defined according to hormone receptor (HR) and HER2 status. HR-positivity was defined as positivity for either the estrogen receptor or the progesterone receptor, and HR/HER2 assessments were based on the American Society of Clinical Oncology/College of American Pathologists guidelines [20, 21]. Treatment-related variables included anthracycline use, trastuzumab use, and radiotherapy. Anthracycline use was defined as the receipt of at least one cycle of doxorubicin plus cyclophosphamide; epirubicin plus cyclophosphamide; or cyclophosphamide, epirubicin, and fluorouracil. Trastuzumab use was defined as the receipt of trastuzumab with or without pertuzumab. Non-anthracycline-based trastuzumab-containing regimens included weekly paclitaxel plus trastuzumab with or without pertuzumab; docetaxel plus cyclophosphamide (TC) plus trastuzumab with or without pertuzumab; and docetaxel, carboplatin, and trastuzumab with or without pertuzumab. Radiotherapy was assessed according to the presence or absence of left-sided irradiation to the breast or chest wall during the perioperative period.

### Statistical analysis

Continuous variables are summarized as medians with the first to third quartiles (Q1–3), and categorical variables as counts and percentages. The cumulative incidences of HF and non-HF MACEs were estimated using the cumulative incidence function (CIF), and between-group differences were assessed using Gray’s test. Breast cancer recurrence and non-cardiovascular event-related death were treated as competing events. Separate competing-risk analyses were performed for HF and non-HF MACEs. Fine–Gray competing-risk regression models were used to estimate subdistribution hazard ratios (sHRs) and 95% confidence intervals (CIs). Because of the limited number of outcome events, the primary multivariable models included four prespecified covariates (age, cardiovascular risk factors, anthracycline use, and trastuzumab use) selected based on their clinical relevance to minimize the risk of overfitting. As a sensitivity analysis, additional multivariable models including left-sided irradiation and clinical stage were also performed to assess the robustness of the findings. All statistical tests were two-sided, and p values < 0.05 were considered statistically significant. Statistical analyses were performed using R version 4.5.2 (R Foundation for Statistical Computing, Vienna, Austria).

## Results

### Patient characteristics

**Fig. 1:**
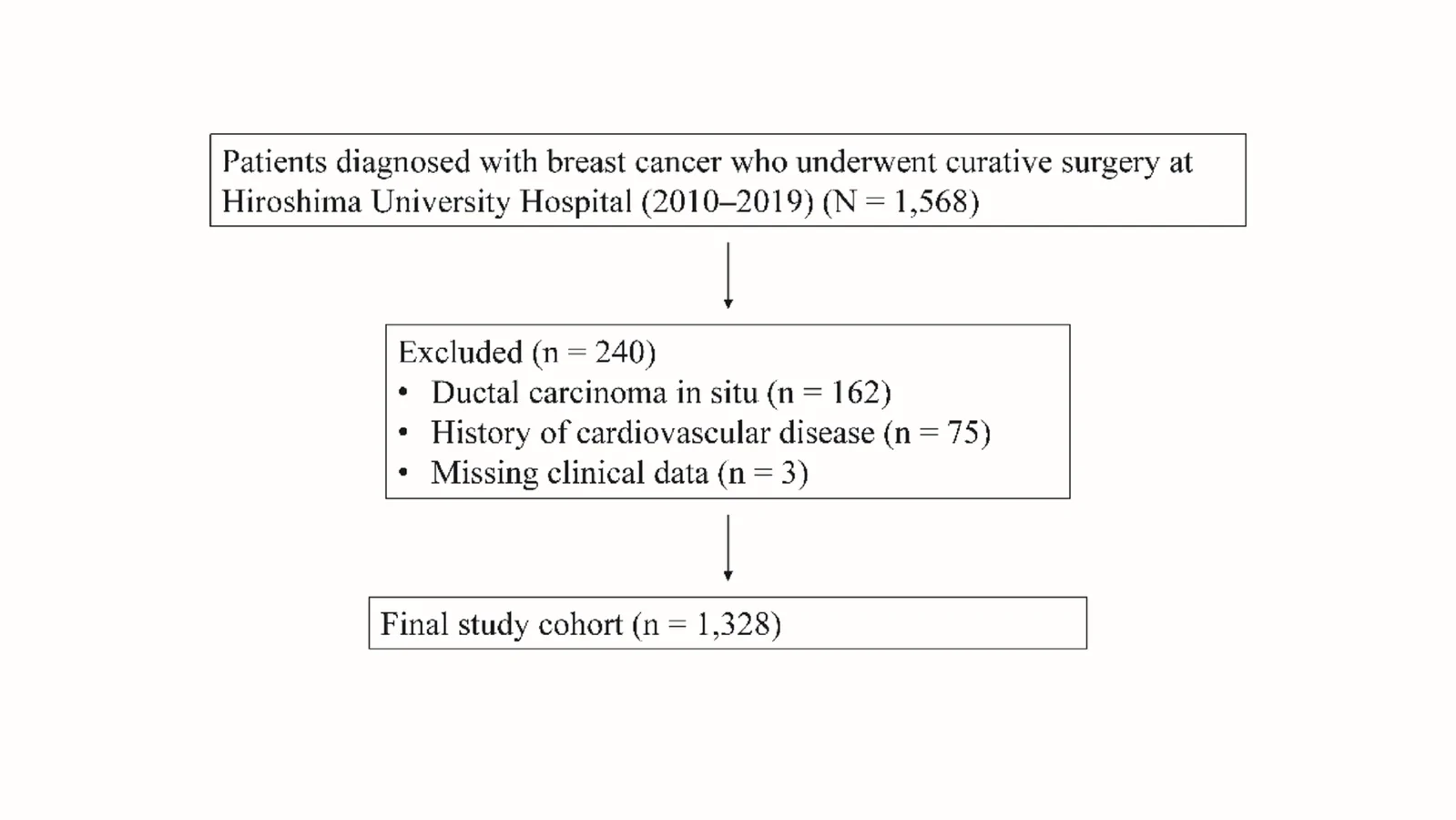
Study flow diagram. Patient selection process for the study cohort

In total, 1,568 patients diagnosed with breast cancer underwent curative surgery at Hiroshima University Hospital from 2010 to 2019. Of these, 1,328 patients were included in the final study cohort (Fig. 1). The median follow-up duration was 7.7 years (Q1–3, 6.1–9.7). The median age was 56.5 years (Q1–3, 46.7–67.5), with 910 patients (68.5%) aged ≤ 65 years. Overall, 398 patients (30.0%) had at least one cardiovascular risk factor (hypertension, diabetes mellitus, or dyslipidemia). Regarding perioperative systemic therapy, 898 patients (67.6%) received neither anthracycline nor trastuzumab, 48 (3.6%) received trastuzumab without anthracycline, 262 (19.7%) received anthracycline without trastuzumab, and 120 (9.0%) received both anthracycline and trastuzumab. These and other patient/tumor characteristics (body mass index, smoking history, tumor stage, tumor subtype, and the receipt of radiotherapy) are summarized in Table 1.

**Table 1:** Patient and treatment characteristics of the study cohort (*N* = 1,328)

Characteristic	Value
**Age**	
Median (Q1–3), years	56.5 (46.7–67.5)
≤ 65 years	910 (68.5%)
> 65 years	418 (31.5%)
**Body mass index**	
Median (Q1–3), kg/m^2^	22.0 (20.0–24.9)
**Comorbidities**	
Hypertension	274 (20.6%)
Diabetes mellitus	73 (5.5%)
Dyslipidemia	234 (17.6%)
**Lifestyle**	
Smoking history (ever)	178 (13.4%)
**Tumor stage**	
I	707 (53.2%)
II	504 (38.0%)
III	117 (8.8%)
**Subtype**	
HR+/HER2−	1,030 (77.6%)
HER2+	185 (13.9%)
Triple-negative	113 (8.5%)
**Systemic therapy**	
Neither anthracycline nor trastuzumab	898 (67.6%)
Trastuzumab-containing therapy without anthracycline	48 (3.6%)
Anthracycline-containing therapy without trastuzumab	262 (19.7%)
Both anthracycline and trastuzumab	120 (9.0%)
**Radiotherapy**	
Left-sided	475 (35.8%)

Values are presented as n (%), unless otherwise indicated

HR, hormone receptor; HER2, human epidermal growth factor receptor 2; Q, quartile

### Clinical events during follow-up

During follow-up, HF occurred in 24 patients (1.8%), while non-HF MACEs occurred in 38 (2.9%). The most frequent non-HF MACE was AF (*n* = 18), followed by stroke (*n* = 13) and MI (*n* = 5). The distribution of clinical events is summarized in Table 2.

**Table 2:** Clinical events during follow-up in patients with breast cancer (*N* = 1,328)

Event	*n* (%)
**Cardiovascular events**	
Heart failure	24 (1.8)
Non-heart failure major adverse cardiovascular events	38 (2.9)
Stroke	13 (1.0)
Myocardial infarction	5 (0.4)
Atrial fibrillation	18 (1.4)
Cardiovascular event-related death	2 (0.2)
**Other clinical events**	
Breast cancer recurrence	139 (10.5)
Non-cardiovascular event-related death	97 (7.3)

Other clinical events were treated as competing events in the analysis of major adverse cardiovascular events

### HF and non-HF MACE analyses

**Fig. 2:**
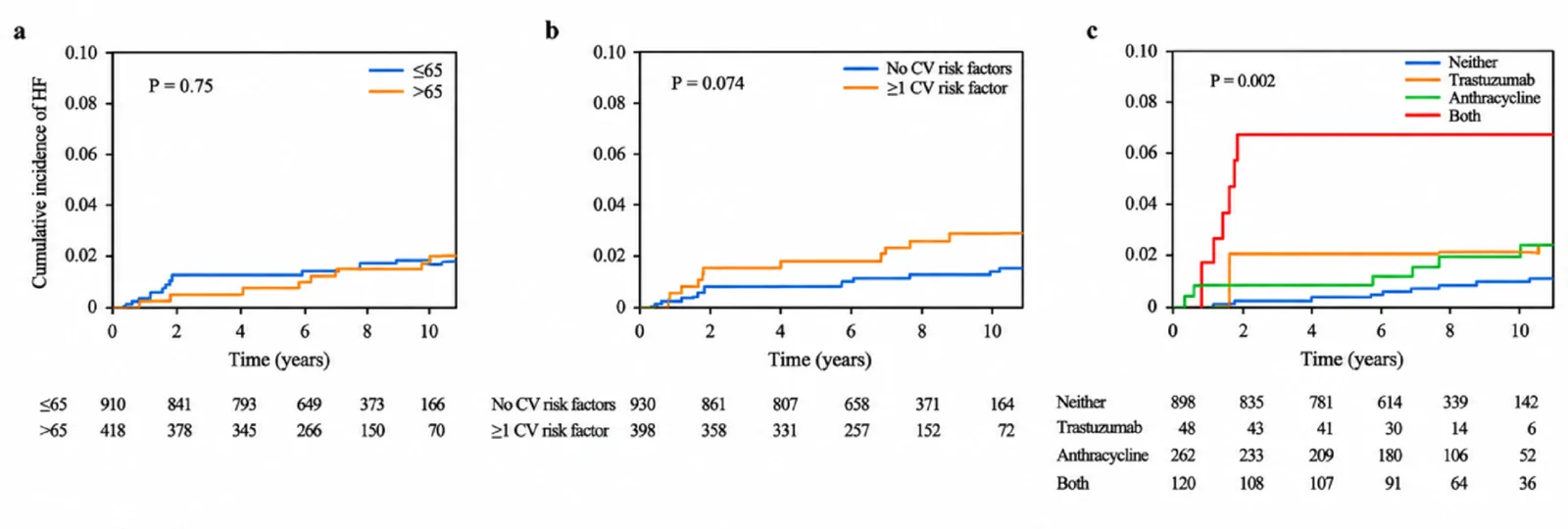
Cumulative incidence of HF according to patient characteristics and systemic therapy. Cumulative incidence functions for HF, estimated using the Fine–Gray competing-risk method. (**a**) Stratified according to age group (≤ 65 vs. >65 years). (**b**) Stratified according to the presence of cardiovascular risk factors (hypertension, diabetes mellitus, or dyslipidemia). (**c**) Stratified according to perioperative systemic therapy exposure. Neither = neither anthracycline nor trastuzumab; Trastuzumab = trastuzumab without anthracycline; Anthracycline = anthracycline without trastuzumab; and Both = both anthracycline and trastuzumab. Breast cancer recurrence and non-cardiovascular event-related death were treated as competing events. Numbers at risk are shown below each panel. P values were calculated using Gray’s test. Abbreviations: HF, heart failure; CV, cardiovascular

The cumulative incidence of HF did not differ significantly according to age (≤ 65 vs. >65 years; Gray’s test, *p* = 0.72) or the presence of cardiovascular risk factors (*p* = 0.074). By contrast, the cumulative incidence of HF differed significantly according to the type of perioperative systemic therapy (*p* = 0.002), with the highest incidence observed in patients treated with both anthracyclines and trastuzumab. The cumulative incidence curves suggested an early increase in HF events in the latter group, whereas increases were less marked among patients treated with anthracyclines alone or trastuzumab alone (Fig. 2). In the multivariable Fine–Gray competing risk regression model, anthracycline use (sHR, 2.67; 95% CI, 1.08–6.65; *p* = 0.034) and trastuzumab use (sHR, 2.52; 95% CI, 1.00–6.33; *p* = 0.050) were independently associated with HF. By contrast, age and the presence of at least one cardiovascular risk factor were not significantly associated with HF (Table 3). The findings were robust in a prespecified sensitivity analysis that additionally adjusted for left-sided radiotherapy and clinical stage. Anthracycline use and trastuzumab use remained independently associated with HF (Supplementary Table S1).

**Table 3:** Fine–Gray competing-risk regression analysis for heart failure

Covariate	sHR (95% CI)	*P* value
Age > 65 years (vs. ≤65 years)	1.11 (0.38–3.25)	0.840
≥ 1 cardiovascular risk factor (vs. none)	2.40 (0.84–6.90)	0.100
Anthracycline use (yes vs. no)	2.67 (1.08–6.65)	0.034
Trastuzumab use (yes vs. no)	2.52 (1.00–6.33)	0.050

sHR, subdistribution hazard ratio; CI, confidence interval

Cardiovascular risk factors were defined as hypertension, diabetes mellitus, or dyslipidemia

Breast cancer recurrence and non-cardiovascular event-related death were treated as competing events

Abbreviations: MACE, major adverse cardiovascular event; HF, heart failure; CV, cardiovascular

**Fig. 3:**
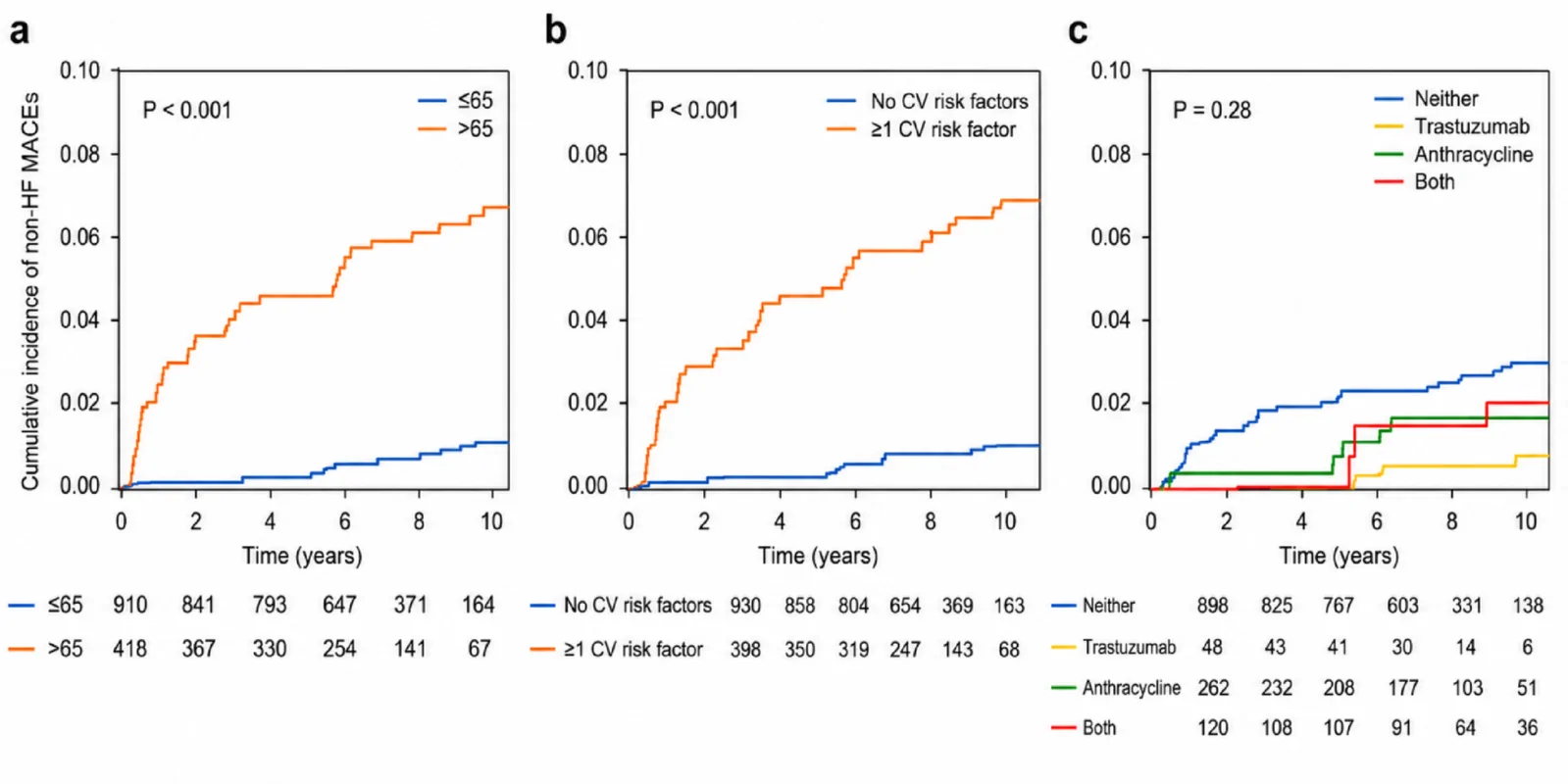
Cumulative incidence of non-HF MACEs according to patient characteristics and systemic therapy. Cumulative incidence functions for non-HF MACEs, estimated using the Fine–Gray competing-risk method. (**a**) Stratified according to age group (≤ 65 vs. >65 years). (**b**) Stratified according to the presence of cardiovascular risk factors (hypertension, diabetes mellitus, or dyslipidemia). (**c**) Stratified according to perioperative systemic therapy exposure. Neither = neither anthracycline nor trastuzumab; Trastuzumab = trastuzumab without anthracycline; Anthracycline = anthracycline without trastuzumab; and Both = both anthracycline and trastuzumab. Breast cancer recurrence and non-cardiovascular event-related death were treated as competing events. Numbers at risk are shown below each panel. P values were calculated using Gray’s test

The cumulative incidence of non-HF MACEs differed significantly according to age (≤ 65 vs. >65 years) and the presence of cardiovascular risk factors (Gray’s test, both *p* < 0.001), with higher incidences observed in older patients and in those with at least one cardiovascular risk factor. By contrast, the cumulative incidence of non-HF MACEs did not differ significantly according to the type of perioperative systemic therapy (*p* = 0.28; Fig. 3). In the multivariable Fine–Gray competing-risk regression model, an age > 65 years (sHR, 3.75; 95% CI, 1.59–8.88; *p* = 0.003) and the presence of cardiovascular risk factors (sHR, 4.02; 95% CI, 1.77–9.10; *p* < 0.001) were independently associated with non-HF MACEs. Anthracycline use, trastuzumab use, the use of left-sided radiotherapy, and clinical stage were not significantly associated with non-HF MACEs (Table 4). The findings were robust in a prespecified sensitivity analysis that additionally adjusted for left-sided radiotherapy and clinical stage. Age > 65 years and the presence of cardiovascular risk factors remained independently associated with non-HF MACEs (Supplementary Table S2).

**Table 4:** Fine–Gray competing-risk regression analysis for non-heart failure major adverse cardiovascular events

Variable	sHR (95% CI)	*P* value
Age > 65 years (vs. ≤65 years)	3.75 (1.59–8.88)	0.003
≥ 1 cardiovascular risk factor (vs. none)	4.02 (1.77–9.10)	< 0.001
Anthracycline use (yes vs. no)	1.15 (0.54–2.44)	0.720
Trastuzumab use (yes vs. no)	0.62 (0.20–1.96)	0.420

sHR, subdistribution hazard ratio; CI, confidence interval

Cardiovascular risk factors were defined as hypertension, diabetes mellitus, or dyslipidemia

Breast cancer recurrence and non-cardiovascular event-related death were treated as competing events

## Discussion

In this retrospective cohort study of patients treated with curative intent for early breast cancer, we evaluated the incidences of and risk factors for HF and non-HF MACEs by using a competing-risk framework. During a median follow-up of 7.7 years, HF occurred in 2.0% of patients, whereas non-HF MACEs occurred in 3.0%. HF was independently associated with anthracycline and trastuzumab exposure, whereas non-HF MACEs were independently associated with older age and the presence of cardiovascular risk factors. These findings indicate that treatment-related cardiotoxicity and the background cardiovascular risk represent distinct mechanisms of cardiovascular morbidity among patients with breast cancer.

Our findings extend previous evidence on treatment-related cardiotoxicity in breast cancer. Although prior clinical trial and observational studies have established anthracycline and trastuzumab use as major causes of cardiac dysfunction and HF [5, 6, 22], our results further suggest that the HF risk in this setting is driven more strongly by treatment exposure than by age or cardiovascular risk factors. Notably, the cumulative incidence of HF increased most prominently in patients treated with both anthracyclines and trastuzumab, particularly within the first 2 years after diagnosis, whereas increases were more modest in those treated with either agent alone. This finding is consistent with findings from large adjuvant trials such as BCIRG-006 and N9831, which demonstrated higher rates of cardiac dysfunction with trastuzumab-containing regimens, especially when combined with anthracycline-containing chemotherapy [23, 24]. By contrast, population-based analyses have revealed that anthracycline therapy alone is associated with only a modest increase in the HF risk over no chemotherapy [22]. Taken together, these findings suggest that the combination of anthracyclines and trastuzumab may be the principal driver of the short-term risk of HF in patients undergoing curative treatment for early breast cancer. Clinically, this supports the closer cardiac surveillance of patients receiving combined cardiotoxic therapy regardless of patient-related risk factors at diagnosis.

Although heart failure generally increases with age in the general population, age was not independently associated with heart failure in the present study. One possible explanation is that heart failure events in this cohort primarily reflected cancer therapy-related cardiac dysfunction, for which exposure to anthracyclines and HER2-targeted therapies is considered the principal determinant according to contemporary cardio-oncology guidelines. In routine clinical practice, these cardiotoxic agents may also be used more cautiously in older patients because of concerns regarding treatment-related cardiotoxicity, which could have attenuated the association between chronological age and heart failure [25]. Furthermore, patients with a history of major cardiovascular events were excluded from the present study, and only patients with early breast cancer undergoing curative-intent treatment were included. Consequently, the older patients enrolled in this cohort were likely to represent a relatively healthy population with a lower baseline cardiovascular risk than the general elderly population [26]. Finally, although cardiovascular risk factors were not independently associated with heart failure, patients with these risk factors showed a trend toward a higher incidence of heart failure, suggesting that baseline cardiovascular vulnerability may still have contributed to the development of cancer therapy-related cardiac dysfunction.

Importantly, cardiovascular outcomes after breast cancer treatment are not limited to HF [10]. In our cohort, non-HF MACEs—stroke, MI, AF, and cardiovascular death—were at least as common as HF and were associated primarily with older age and cardiovascular risk factors rather than treatment exposure. Anthracycline and trastuzumab use were not significantly associated with these events, despite being associated with HF. This pattern is consistent with that in previous population-based studies showing that MACEs after diagnosis of early breast cancer are largely driven by pretreatment cardiovascular risk profiles [27]. AF was included as a component of non-HF MACEs because patients with cancer reportedly have an increased risk of AF compared with the general population [28]. In addition, breast cancer recurrence was treated as a competing event because it substantially alters the subsequent systemic therapy and prognosis. Together, these findings suggest that long-term cardiovascular morbidity among patients with breast cancer is shaped not only by treatment-related cardiotoxicity but also by the patient’s underlying cardiovascular risk burden.

These findings may have implications for treatment selection in early breast cancer. For HER2-positive disease, our results support current efforts to avoid anthracycline exposure when effective alternatives are available, because the HF risk was concentrated in patients exposed to both anthracyclines and trastuzumab. This is consistent with recent trials that demonstrated the efficacy of anthracycline-free regimens among patients receiving modern anti-HER2 therapy [29, 30]. By contrast, for HER2-negative disease, anthracyclines should not be considered uniformly avoidable. The ABC trials suggested that anthracycline-containing regimens provide a greater benefit than TC for patients with higher-risk disease [31], and more recent genomic analyses have indicated that patients with genomically high-risk tumors may derive a meaningful benefit from anthracycline-based chemotherapy [32]. In our cohort, anthracycline exposure without anti-HER2 therapy was associated with a more modest HF risk than combined anthracycline–trastuzumab exposure. Taken together, these findings support an individualized approach in which anthracycline use is minimized in HER2-positive disease but selectively considered in HER2-negative disease after balancing the oncologic benefit against the patient’s baseline cardiovascular risk.

This study has several limitations. First, its retrospective, single-center design might have limited the generalizability of the findings. Second, cardiovascular events were identified based on medical records without formal event adjudication, which might have resulted in misclassification. Third, few cardiovascular events occurred; therefore, the multivariable models were restricted to clinically relevant variables selected a priori to minimize the risk of overfitting. However, given the limited number of events relative to the number of covariates included, there remains a possibility of model overfitting and reduced stability of the estimates; thus, the results should be interpreted with caution. Future studies are warranted to validate these findings in larger, multicenter cohorts and to determine whether integrated risk-based cardio-oncology strategies can improve long-term cardiovascular outcomes.

In conclusion, HF and non-HF MACEs may represent distinct patterns of cardiovascular morbidity among patients with breast cancer. HF was more closely associated with cancer therapy, whereas non-HF MACEs were more strongly related to patient-related cardiovascular risk factors. These findings suggest that cardio-oncology strategies for patients with breast cancer should consider both treatment-related cardiotoxicity and their long-term cardiovascular risk.

## Data Availability

The datasets generated and analyzed during the current study are available from the corresponding author upon reasonable request.
